# Antioxidant activity of Jinxuan tea polysaccharide and its protective effect on intestinal injury induced by transport stress in chicks

**DOI:** 10.3389/fvets.2025.1610218

**Published:** 2025-06-25

**Authors:** Xueyan Sun, Qiaoyi Zhou, Jinjing Gao, Shujuan Liu, Feike Zhao, Caijin Ling, Binghu Fang

**Affiliations:** ^1^National Reference Laboratory of Veterinary Drug Residues, College of Veterinary Medicine, South China Agricultural University, Guangzhou, China; ^2^Guangdong Provincial Key Laboratory of Tea Plant Resources Innovation and Utilization, Tea Research Institute, Guangdong Academy of Agricultural Sciences, Guangzhou, China

**Keywords:** tea polysaccharide, antioxidant activity, transport stress, intestinal injury, intestinal flora

## Abstract

**Introduction:**

Transport stress (TS) is unavoidable for livestock and poultry in modern agriculture, which not only affects animal welfare, but also to the quality of meat products. Tea polysaccharide (TPS), one of the major bioactive components of tea leaves, has been shown to have anti-stress effects. However, the antagonistic effect of TPS on TS-induced intestinal damage in chicks is unclear.

**Methods:**

The free radical scavenging and reducing abilities of TPS were determined by chemical methods. RAW264.7 macrophages were stimulated by H_2_O_2_ to construct cell oxidative damage model, and the effects of TPS on cell viability and antioxidant enzyme activity were determined. A chick transport stress model was established using the shaker to investigate the effect of TPS on intestinal damage in chick caused by transport stress.

**Results:**

*In vitro* test results showed that TPS had good free radical scavenging ability and reducing ability. Furthermore, TPS could significantly alleviate cell oxidative damage induced by H_2_O_2_. *In vivo* test results showed that the TS-induced intestinal pathological damage and oxidative damage were markedly alleviated by TPS. Furthermore, TS resulted in the elevated expression of heat shock factors, disrupted expression of heat shock proteins, and similarly disrupted expression of aquaporins in chicks. Surprisingly, chicks treated with TPS showed a significant decrease in the expression of heat shock factors and significant alleviation of the dysregulated expression of heat shock proteins and aquaporins. Analysis of the intestinal flora showed that TS resulted in reduced intestinal flora diversity and altered flora structure in chicks, whereas the intestinal flora was normalized after TPS intervention.

**Discussion:**

TPS has good antioxidant activity and significantly ameliorate TS-induced intestinal damage in chicks, suggesting that TPS could be developed and utilized as a feed additive.

## 1 Introduction

With the increasing scale and intensity of the livestock and poultry industry, the necessity for seed introduction and export becomes paramount. Consequently, the use of vehicles for off-site transportation has become an indispensable aspect of the breeding industry. During the transportation process, animals might be exposed to a range of stressors, including fluctuations in temperature, fasting, water deprivation, noise, and other factors (1). Transport stress (TS) often has adverse effects, including physical damage to the body, performance degradation, and even death (2). The immature nature of the newborn chick’s body renders it susceptible to stressors encountered during transportation. It has been demonstrated that TS not only disrupts the internal environmental homeostasis of chicks (3), but also causes varying degrees of damage to vital organs, such as the heart, liver, and brain (4–6).

The intestine is the primary organ that responds to stress, which is prone to mucosal damage, slow peristalsis, and other functional disorders when experiencing stress (7, 8). Furthermore, stress can result in a reduction in the diversity and abundance of the intestinal flora, thus affecting the normal development of intestinal metabolism and digestive absorption (9). In addition, TS was observed to cause oxidative damage to jejunal tissue in pigs (10) and a significant increase in small intestinal cell apoptosis in goats (11). However, there is a lack of information on the effects of TS on the intestine of newly hatched chicks.

Tea is an agricultural product derived from the fresh leaves and buds of the tea tree. Tea contains a variety of bioactive components, including polyphenols, theanines, alkaloids, and polysaccharides (12). Tea polysaccharide (TPS), the primary active component of tea, is a high-molecular-weight polymer with a complex structure. Studies have demonstrated that TPS exhibits a range of biological activities, including antioxidant, anti-inflammatory, anti-tumor, and hypoglycemic effects (13–16). Yu extracted a novel acidic polysaccharide, HTP-1, from Hawk tea. The administration of HTP-1 was observed to not only attenuate jejunal injury and improve immune organ indicators in mice, but also increase the abundance of beneficial bacteria (17). Given its multiple biological activities, TPS has significant potential as a natural product in the development of functional feeds. Jin Xuan TPS is an acidic heteropolysaccharide, which was demonstrated to ameliorate metabolic disorders and bolster immune function in immunosuppressed mice (18). However, there is a paucity of research examining the antagonistic effects of TPS on TS.

In this study, we investigated the antioxidant activity of Jin Xuan TPS and evaluated its antagonistic effect on TS-induced intestinal damage in chicks in terms of pathological damage, oxidative damage, heat shock response (HSR), aquaporins (AQPs) and intestinal flora. The aim is to provide a reference basis for the development and utilization of TPS as a feed additive.

## 2 Materials and methods

### 2.1 Materials

TPS was extracted in the pre-laboratory stage. The crude polysaccharide was extracted from fresh Jin Xuan tea leaves using ethanol precipitation, followed by deproteinization (Sevage method) and degreasing (petroleum ether). Initial purification was performed using AB-8 macroporous resin and dialysis. Further purification was achieved via DEAE anion-exchange chromatography and Sephacryl S-400 HR gel filtration. The final product was desalted by dialysis and lyophilized to obtain purified TPS. The Mw (Weight-average molecular weight) of TPS is 478.75 kDa with a purity of 68.89%, which consisted mainly of Ara (Arabinose), Gal (Galactose), Gal-UA (Galacturonic acid), Glc (Glucose), Rha (Rhamnose), Man (Mannose), Glc-UA (Glucuronic acid), Xyl (Xylose), and Fuc (Fucose) with the molar ratios of 4.86:5.36:0.21:1.00:1.60:0.49:0.59:0.30:0.13 (19).

### 2.2 Antioxidant activity

TPS and ascorbic acid (VC) were prepared as 5, 4, 3, 2, 1 and 0.5 mg/mL aqueous solutions, respectively. After weighing 23.64 mg DPPH (2,2-diphenyl-1-picrylhydrazyl) dissolved in a small amount of methanol, it was transferred to a 500 mL volumetric flask, and 50% ethanol was added to fix the volume to obtain 120 μmol/L DPPH solution, which was stored under protection from light. The reaction was carried out by adding 0.1 mL of sample solution and 1.9 mL of DPPH solution. The absorbance change was detected at 525 nm after standing for 20 min at room temperature. The clearance was calculated according to the following formula:


$$\text{DPPH free radical scavenging}\;(\%)=(1-\frac{A-B}{A0})\times 100\%$$

Where A0 is the absorbance of the blank (50% ethanol instead of the sample), A is the absorbance of the sample, and B is the absorbance of the control (50% ethanol instead of DPPH solution).

The ABTS [2,2′-Azinobis-(3-ethylbenzthiazoline-6-sulphonate)] free radical scavenging ability, hydroxyl free radical inhibiting ability and iron ion reducing ability of TPS were determined by kit following the supplier’s guidelines (Nanjing Jiancheng Bioengineering Institute Co., Ltd., Nanjing, China).

### 2.3 Effects of TPS on RAW264.7 macrophages under oxidative stress

#### 2.3.1 Cell culture

RAW264.7 macrophages is purchased from Wuhan Punosai Life Technology Co., LTD. RAW264.7 macrophages were cultured in DMEM (Dulbecco’s modified eagle medium) at 37°C with 5% CO_2_. When the cell confluence reached 85%, it was digested from the plate and washed three times with PBS for subsequent experiments.

#### 2.3.2 Establishment of oxidative damage model of RAW264.7 macrophages

After the cells were inoculated in 96-well plate for 24 h, added H_2_O_2_ solutions with different concentrations (final concentrations of 100, 200, 300, 300, 400, 500, 600, and 700 μmol/L) and incubated for another 24 h, respectively. Then, the medium was removed and fresh medium containing 10% CCK-8 solution was added into the plate for 4 h to measure the cell viability.

#### 2.3.3 Cell viability analysis

After the cells were inoculated in 96-well plate for 24 h, added new medium containing TPS solution (final concentration was 4 μg/mL) and incubated for another 24 h. Subsequently, new medium containing H_2_O_2_ (finally concentration was 300 μmol/L) replaced the solution for 24 h. Then, the medium was removed and fresh medium that containing 10% CCK-8 solution was added into the plate for 4 h to evaluate the cell viability.

#### 2.3.4 Antioxidant enzyme activity assay

After the cells were inoculated in 96-well plate for 24 h, added new medium containing TPS solution (final concentration was 4 μg/mL) and incubated for another 24 h. Subsequently, new medium containing H_2_O_2_ (finally concentration was 300 μmol/L) replaced the solution for 24 h. Then, the medium was removed, washed 3 times with PBS, and the cells were collected. The activity of the total antioxidant capacity (T-AOC) and total superoxide dismutase (T-SOD) in the cells was determined by ELISA (MSD BioTech Co., Ltd. Huhan, China). Glutathione peroxidase (GPx) activities in cells were detected using assay kits following the supplier’s guideline (Beyotime Biotechnology Co., Ltd., Shanghai, China).

### 2.4 Effect of TPS on TS-induced intestinal damage in chicks

#### 2.4.1 Ethics statement

Every procedure and protocol involving animals was approved by the Experimental Animal Ethics Committee of South China Agricultural University on July 12, 2023 (approval ID: 2023A019).

#### 2.4.2 Animal and trial design

We developed a simulated transport model for newborn chicks based on the experiments of Dadgar and Chen (20, 21). The study utilized a total of 40 one-day-old chicks (Ephedra chicken, Guangdong Wen’s Dahuanong Biotechnology Co., Ltd., Yun-fu, China). The chicks were randomly divided into five groups: a control group (Con), a simulated transport stress group (TS), a TS plus low dose TPS group (L-TPS), a TS plus medium-dose TPS group (M-TPS), and a TS plus high-dose TPS group (H-TPS), with each group comprising eight chicks. We designed the administration dose of TPS based on the experiments of Zhou and Lan (18, 22). One hour before the commencement of the transportation process, the chicks in Con and TS were administered 0.3 mL of deionized water, while the chicks in the L-TPS, M-TPS, and H-TPS received TPS at doses of 200, 400, and 600 mg/kg body weight (b.w.) respectively. The chicks in Con were maintained at 28°C for 5 h within a warm box, while the remaining chicks were subjected to a simulated transportation process on a shaker with an oscillation frequency of 60–80 r/min at 32°C. During the experimental period, the chicks in each group were not provided with food or water. At the conclusion of simulated transport, all chicks were euthanized with carbon dioxide, and jejunal tissues were promptly collected and preserved in liquid nitrogen for subsequent experimentation. A portion of the jejunal tissue was stored in a 4% paraformaldehyde solution. The cecum feces were collected and stored in a − 80°C refrigerator for subsequent analysis.

#### 2.4.3 Histopathological observation

Adequately fixed jejunal tissues were dehydrated through a concentration gradient of ethanol, followed by embedding, sectioning, and hematoxylin and eosin (H&E) staining. After staining, the histopathological changes in the jejunum were observed under a microscope. Villi length and crypt depth were measured using ImageJ software (National Institutes of Health, Bethesda, MD, USA).

#### 2.4.4 Antioxidant enzyme activity assay

T-AOC, T-SOD and GPx activities, and the malondialdehyde (MDA) content in chick jejunum were detected using their respective assay kits following the supplier’s guidelines (Beyotime Biotechnology Co., Ltd., Shanghai, China).

#### 2.4.5 Total RNA extraction and quantitative real-time reverse transcription PCR

Jejunal tissue (50 mg) was obtained and processed using the RNAiso Plus reagent (Takara Inc., Dalian, China) in accordance with the manufacturer’s instructions. The resulting total RNA was quantified using an ultra-micro spectrophotometer. Then, 1 μg of total RNA was reverse transcribed into cDNA using an RT reagent Kit (Takara Inc., Dalian, China). The resultant cDNA was then amplified using SYBR qPCR Master Mix (Vazyme Biotech Co. Ltd., Nanjing, China) on an ABI Quant Studio 5 Flex PCR instrument (Applied Biosystems, Foster City, CA, USA) for the qPCR reactions. The primers utilized in the procedure are detailed in Table 1, with *β-actin* serving as the internal reference gene. The 2^-ΔΔCT^ method was employed to determine the relative mRNA expression levels.

**Table 1:** The sequences of oligonucleotide primers.

Gene name	Forward primer	Reverse primer	NCBI accession no.
*β-actin*	TATTGCTGCGCTCGTTGTTG	TAGATGGGAACACAGCACGG	NM_205518.1
*HSF1*	CAGGGAAGCAGTTGGTTCACTACACG	CCTTGGGTTTGGGTTGCTCAGTC	L-06098.1
*HSF2*	CGCTGCTCGCATTCCT	TGTGGCCTCACTTGCTTC	NM_001167764.3
*HSF3*	TCCACCTCTCCTCTCGGAAG	CAACAGGACTGAGGAGCAGG	NM_001305041.2
*HSP27*	ACACGAGGAGAAACAGGATGAG	ACTGGATGGCTGGCTTGG	NM_205290.1
*HSP40*	GGGCATTCAACAGCATAGA	ATTCCATCCCCAAGTTTAGG	NM_001199325.1
*HSP60*	AGCCAAAGGGCAGAAATG	TACAGCAACAACCTGAAGACC	NM_001012916.1
*HSP70*	CGGGCAAGTTTGACAA	TTGGCTCCCACCCTATCTCT	NM_001006685.1
*HSP90*	TCCTGTCCTGGCTTTAGTTT	AGGTGGCATCTCCTCGGT	NM_001109785.1
*HSP110*	CTCCTGAAGAGAAGCCACGA	CCATCAAAGTTCCTTCCGCCT	NM_001159698
*AQP1*	ATGTTCTGGAGGGCGGT	TGAGTTGCTGATGTCCCGT	NM_001039453.1
*AQP3*	GGCATTTTGATTGCAGGCCA	GAGCCAAGAAACACATGGCG	XM_424500.7
*AQP7*	CAATCCGTAGCAACTCCGTCAG	TGAGTGAGTGTGATGGCAGCAT	XM_025144776.1
*AQP11*	GGAGGTTGGAGATGGCGGAAT	GCATGGTGATGAGGAGAGCAA	XM_015280878.2

#### 2.4.6 Determination of the intestinal flora

A high-throughput sequencing analysis of the 16S rDNA was conducted on the cecum contents of three randomly selected chick. Genomic DNA was extracted from cecum feces using a DNA extraction kit (Omega Biotek Guangzhou Ltd., Guangzhou, China) and the extracted genomic DNA was detected using 1% agarose gel electrophoresis. The V3-V4 region of 16S rDNA was amplified using Fastpfu DNA Polymerase (TransGen Biotech Co., Ltd., Beijing, China) with the following primer sequences: 338F (ACTCCTAC-GGGGAGGCAGCAG) and 806R (GGACTACHVGGGTWT CTAAT). Purification of the DNA was performed using a DNA Gel Extraction Kit (Heyi Biotech Co., Ltd., Beijing, China). Libraries were then constructed and sequenced on the Illumina NovaSeq PE250 platform (San Diego, CA, USA). Finally, the sequencing reads and operational taxonomic units (OTUs) were analyzed. The impact of TPS on the intestinal flora of TS-treated chicks was evaluated in terms of alpha diversity, beta diversity, and flora structure.

### 2.5 Statistical analysis

The data were analyzed using SPSS 21.0 (IBM Corp., Armonk, NY, USA), with one-way ANOVA, and the statistical difference was compared using the Tukey test. A *p* value less than 0.05 was considered statistically significant, and the results are expressed as the mean ± standard deviation. Subsequently, the data were plotted using GraphPad Prism 9.5 (GraphPad Inc., San Diego, CA, USA).

## 3 Results

### 3.1 Antioxidant activity of TPS

**Figure 1:**
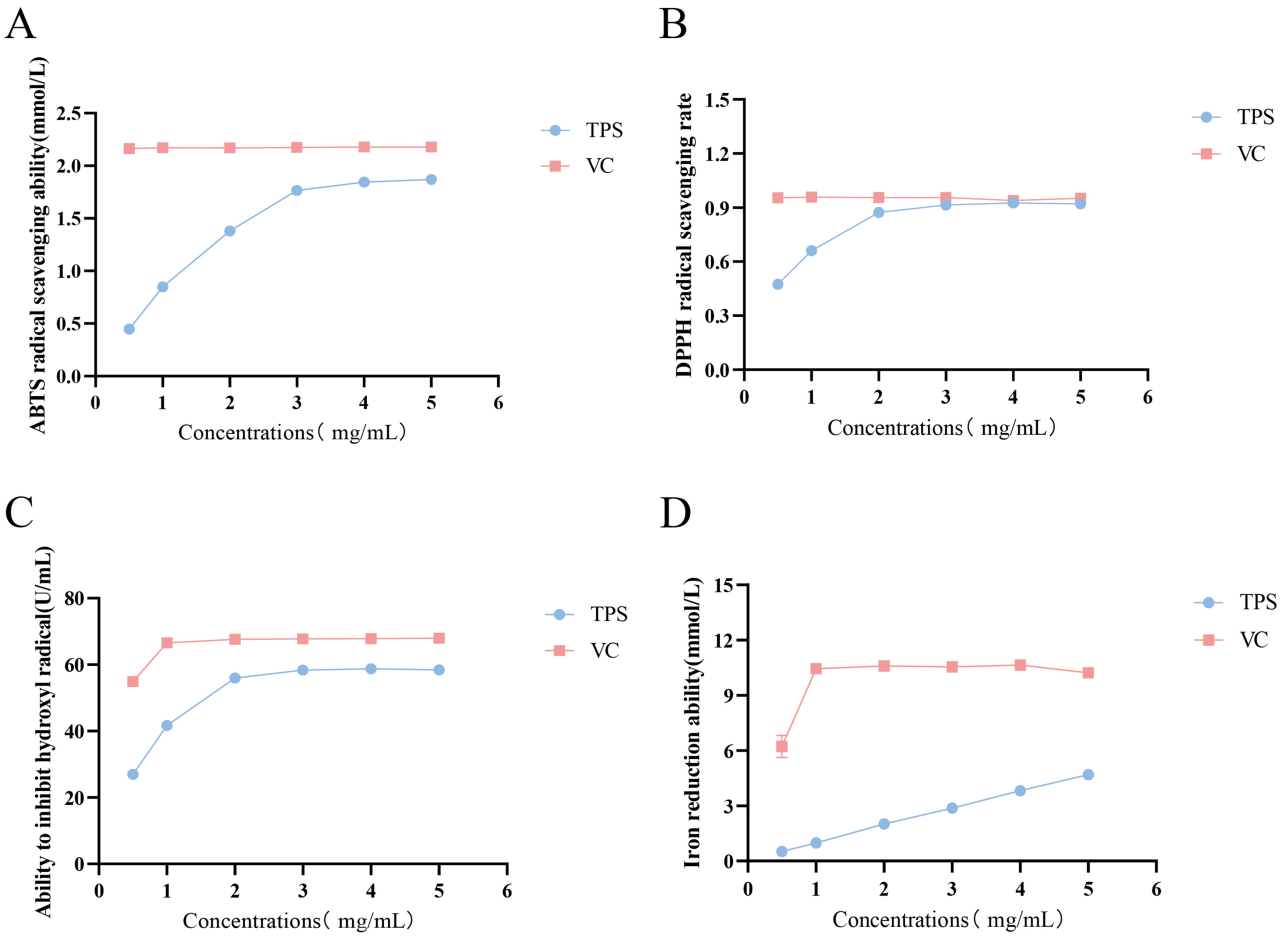
Antioxidant activity of TPS. **(A)** ABTS free radical scavenging ability. **(B)** DPPH free radical scavenging ability. **(C)** Hydroxyl free radical inhibiting ability. **(D)** Iron ion reducing ability.

As shown in Figure 1A, the scavenging capacity of TPS for ABTS radicals increased with increasing concentration, reaching a maximum of 1.87 mmol/L at 4 mg/mL. As shown in Figure 1B, the scavenging capacity of TPS for DPPH radicals increased with increasing concentration, and its scavenging rate of DPPH radicals reached a maximum of 91.42% at 3 mg/mL, which was comparable to the effect of the same concentration of VC. As shown in Figure 1C, the hydroxyl radical inhibition ability of TPS increased with increasing concentration, reaching the maximum of 58.74 U/mL at 3 mg/mL. As shown in Figure 1D, the iron ion reducing ability of TPS was always positively correlated with its concentration in the concentration range of 0.5–5 mg/mL.

### 3.2 Effects of TPS on RAW264.7 macrophages under oxidative stress

#### 3.2.1 Establishment of oxidative damage model of cells

**Figure 2:**
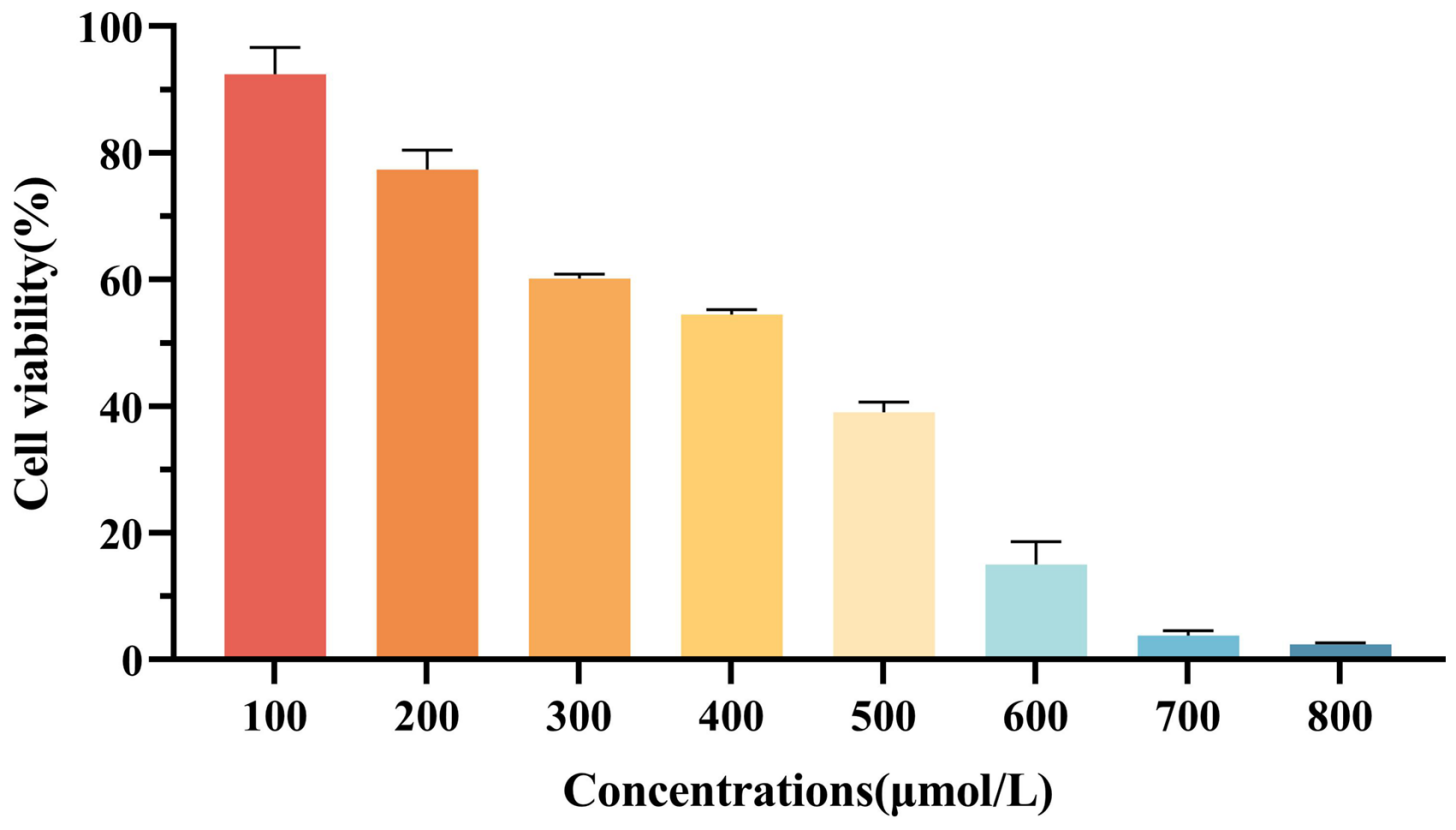
Effect of H_2_O_2_ on the viability of RAW264.7 macrophages.

As shown in Figure 2, when H_2_O_2_ concentration was 300 μmol/L, the cell viability was (60.18 ± 0.70) %, indicating that oxidative stress occurred in cells but no irreversible damage occurred. Therefore, 300 μmol/L H_2_O_2_ was selected for subsequent tests.

#### 3.2.2 Effect of TPS on the viability of cells induced by H_2_O_2_

**Figure 3:**
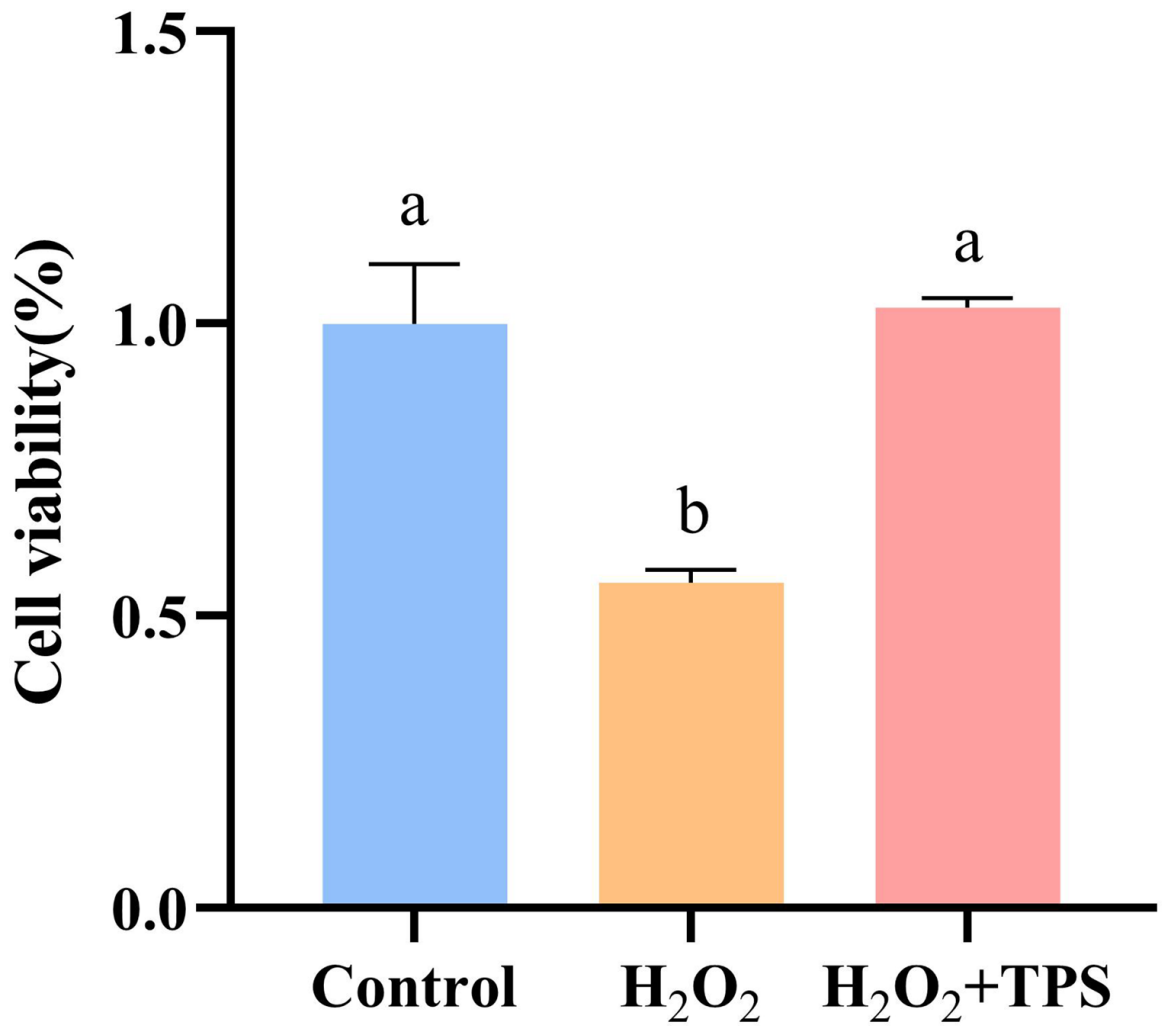
Effect of TPS on the viability of RAW264.7 macrophages induced by H_2_O_2_. Distinct letters indicate significant differences by one way analysis of variance (ANOVA) (*p* < 0.05).

The effect of TPS on cell viability induced by H_2_O_2_-induced oxidative damage is shown in Figure 3. Compared with the control group, the cell viability decreased significantly after H_2_O_2_ stimulation (*p* < 0.05), which was (55.67 ± 0.02) %. Compared with H_2_O_2_ group, the cell viability after adding TPS was significantly increased (*p* < 0.05), which was (102.76 ± 0.02) %. Furthermore, there was no significant difference compared with the control group (*p* > 0.05). The results showed that TPS could significantly alleviate the apoptosis induced by H_2_O_2_.

#### 3.2.3 Effect of TPS on antioxidant enzyme activity in cells induced by H_2_O_2_

**Figure 4:**
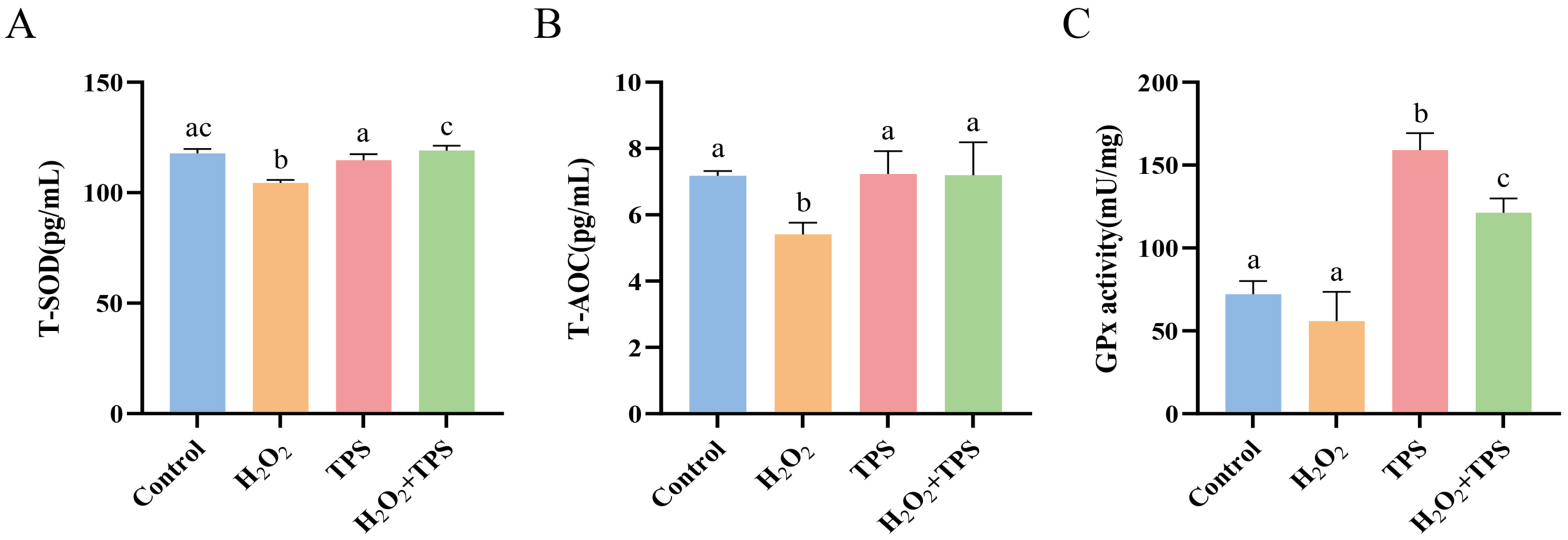
Effect of TPS on the activity of **(A)** T-SOD, **(B)** T-AOC and **(C)** GPx in of RAW264.7 macrophages induced by H_2_O_2_. Distinct letters indicate significant differences by one way analysis of variance (ANOVA) (*p* < 0.05).

As shown in Figure 4. Compared with the control group, T-SOD and T-AOC activities in the cells of the H_2_O_2_ group were significantly reduced (*p* < 0.05), and there was a tendency for GPx activity to decrease (*p* > 0.05). This indicated that H_2_O_2_ induced oxidative damage in RAW264.7 macrophages. T-SOD, T-AOC, and GPx activities were significantly increased in the H_2_O_2_ + TPS group compared to the H_2_O_2_ group (*p* < 0.05). In addition, the T-SOD and T-AOC activities in the TPS group were not significantly different from those in the control group (*p* > 0.05), and the GPx activity in the TPS group was significantly higher than that in the control group (*p* < 0.05). The results indicated that TPS significantly alleviated H_2_O_2_-induced oxidative damage in cells.

### 3.3 Effect of TPS on TS-induced intestinal damage in chicks

#### 3.3.1 Effect of TPS on TS-induced intestinal pathological damage in chicks

**Figure 5:**
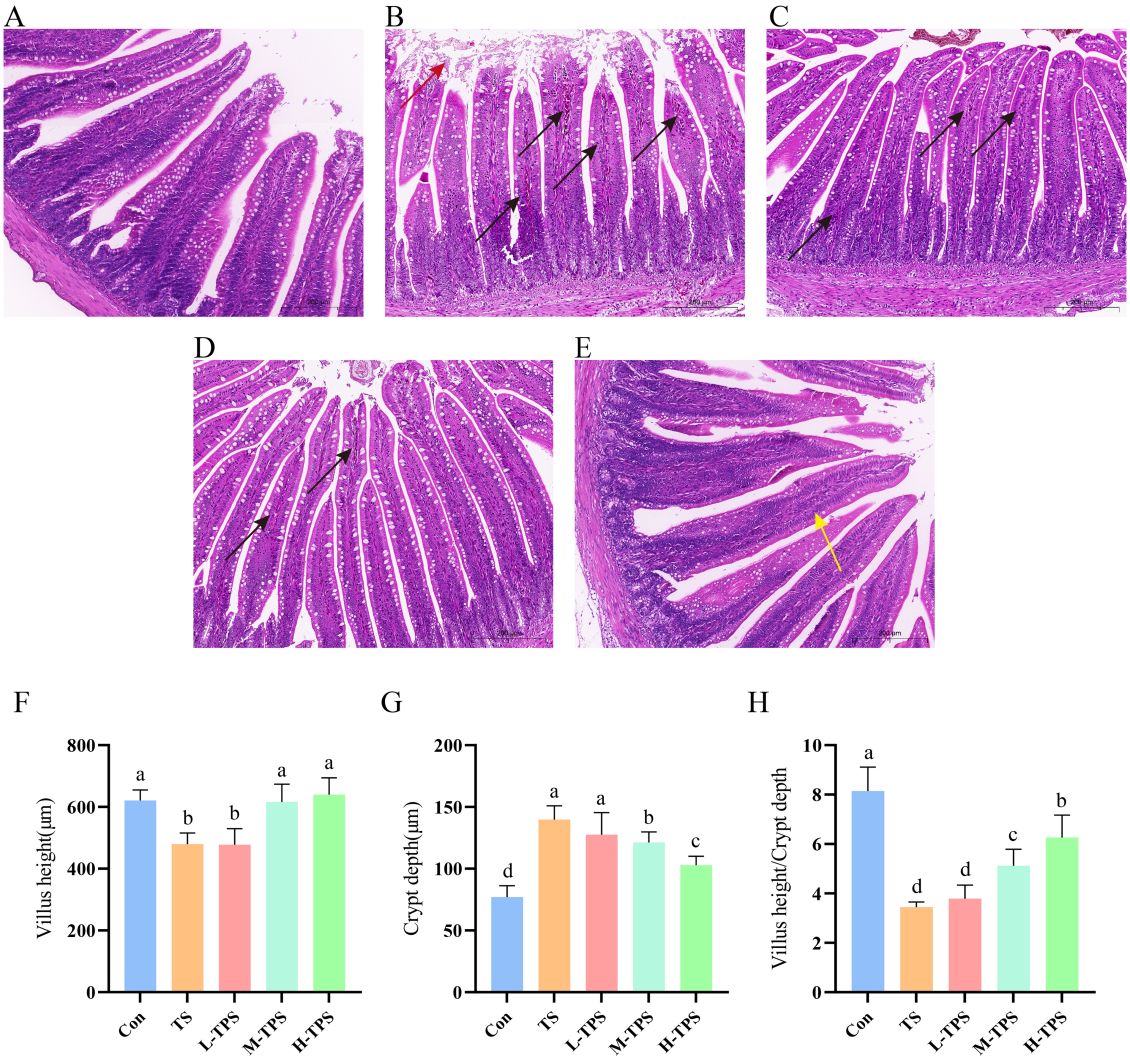
Effect of TPS on TS-induced intestinal pathological damage in chicks. **(A–E)** Hematoxylin and eosin (H&E) staining of jejunum tissue sections of **(A)** Con, **(B)** TS, **(C)** L-TPS, **(D)** M-TPS and **(E)** H-TPS. The red arrows indicate epithelial cell detachment. The black arrows indicate inflammatory cell infiltration. The yellow arrows indicate a decrease in cup cells. **(F–H)** Quantification of the **(F)** villi height, **(G)** crypt depth, and **(H)** villi height/crypt depth ratio in the jejunum. Distinct letters indicate significant differences by one way analysis of variance (ANOVA) (*p* < 0.05).

As shown in Figures 5A–E, the jejunal tissues in the Con were clear and intact in all layers, with long intestinal villi and no obvious lesions. In the TS, some epithelial cells of the intestinal villi were detached and there was infiltration of inflammatory cells in the lamina propria. In the L-TPS and M-TPS, the intestinal villi were intact with well-defined cup cells, and there was minimal infiltration of inflammatory cells in the lamina propria. In the H-TPS, the intestinal villi were highly intact with a slight reduction in cup cells. As shown in Figures 5F–H, compared with the Con, the TS had significantly lower intestinal villus height (*p* < 0.05), significantly higher jejunal crypt depth (*p* < 0.05), and significantly lower villus height/crypt depth ratio (*p* < 0.05). Compared with the TS, the M-TPS and H-TPS showed a significant increase in villus height (*p* < 0.05), a significant decrease in crypt depth (*p* < 0.05), and a significant increase in the villus height/crypt depth ratio (*p* < 0.05).

#### 3.3.2 Effect of TPS on TS-induced intestinal oxidative damage in chicks

**Figure 6:**
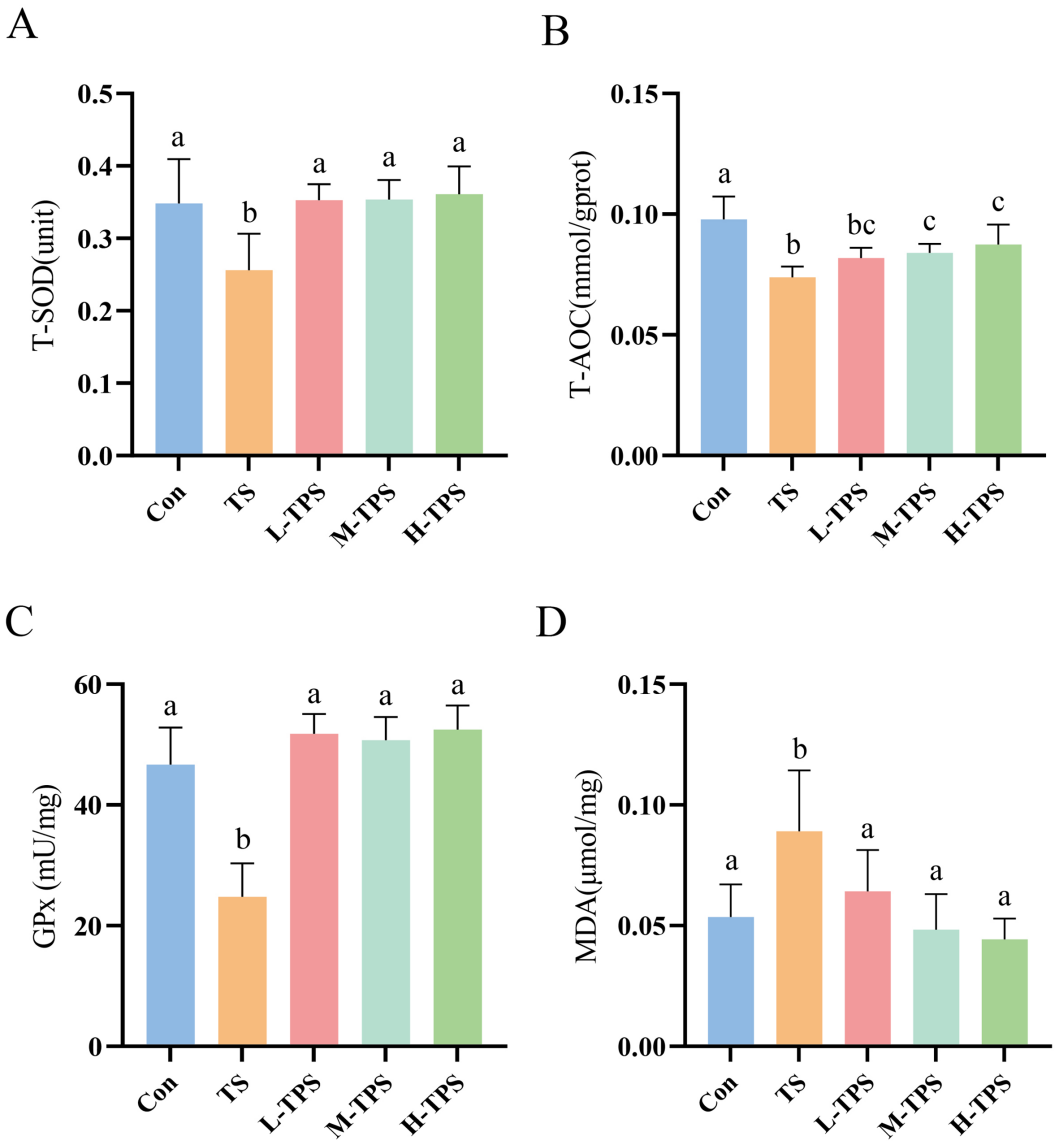
Effect of TPS on TS-induced intestinal oxidative damage in chicks. The contents or activities of **(A)** T-SOD, **(B)** T-AOC, **(C)** GPx and **(D)** MDA. Distinct letters indicate significant differences by one way analysis of variance (ANOVA) (*p* < 0.05).

As shown in Figure 6, compared with the Con, T-AOC, and T-SOD and GPx activities in the TS decreased significantly (*p* < 0.05), and the MDA content increased significantly (*p* < 0.05). In comparison to the TS, the L-TPS, M-TPS, and H-TPS exhibited a notable elevation in T-SOD activity (*p* < 0.05). Additionally, the M-TPS and H-TPS demonstrated a significant enhancement in the T-AOC (*p* < 0.05). The GPx activity was significantly increased in the L-TPS, M-TPS, and H-TPS (*p* < 0.05). Meanwhile, the MDA content was significantly decreased in the L-TPS, M-TPS, and H-TPS relative to the TS (*p* < 0.05).

#### 3.3.3 Effect of TPS on TS-induced heat shock response in chick intestine

**Figure 7:**
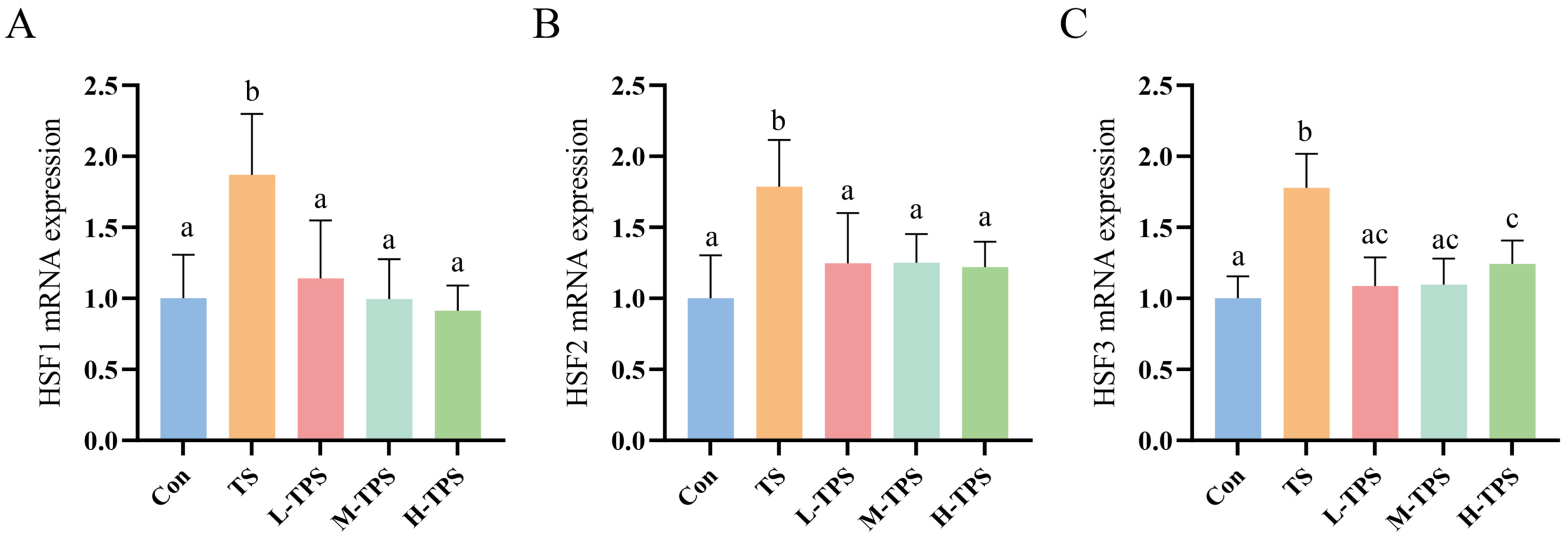
Effect of TPS on TS-induced mRNA expression levels of heat shock factors (HSFs) in the intestine of chicks. The mRNA expression levels of **(A)** *HSF1*, **(B)** *HSF2* and **(C)** *HSF3*. Distinct letters indicate significant differences by one way analysis of variance (ANOVA) (*p* < 0.05).

As shown in Figure 7, significant increases in the mRNA expression levels of *HSF1*, *HSF2*, and *HSF3* were observed in the TS compared with the Con (*p* < 0.05). In comparison with the TS, the L-TPS, M-TPS, and H-TPS all showed a notable reduction in the mRNA expression levels of *HSF1*, *HSF2*, and *HSF3* (*p* < 0.05).

**Figure 8:**
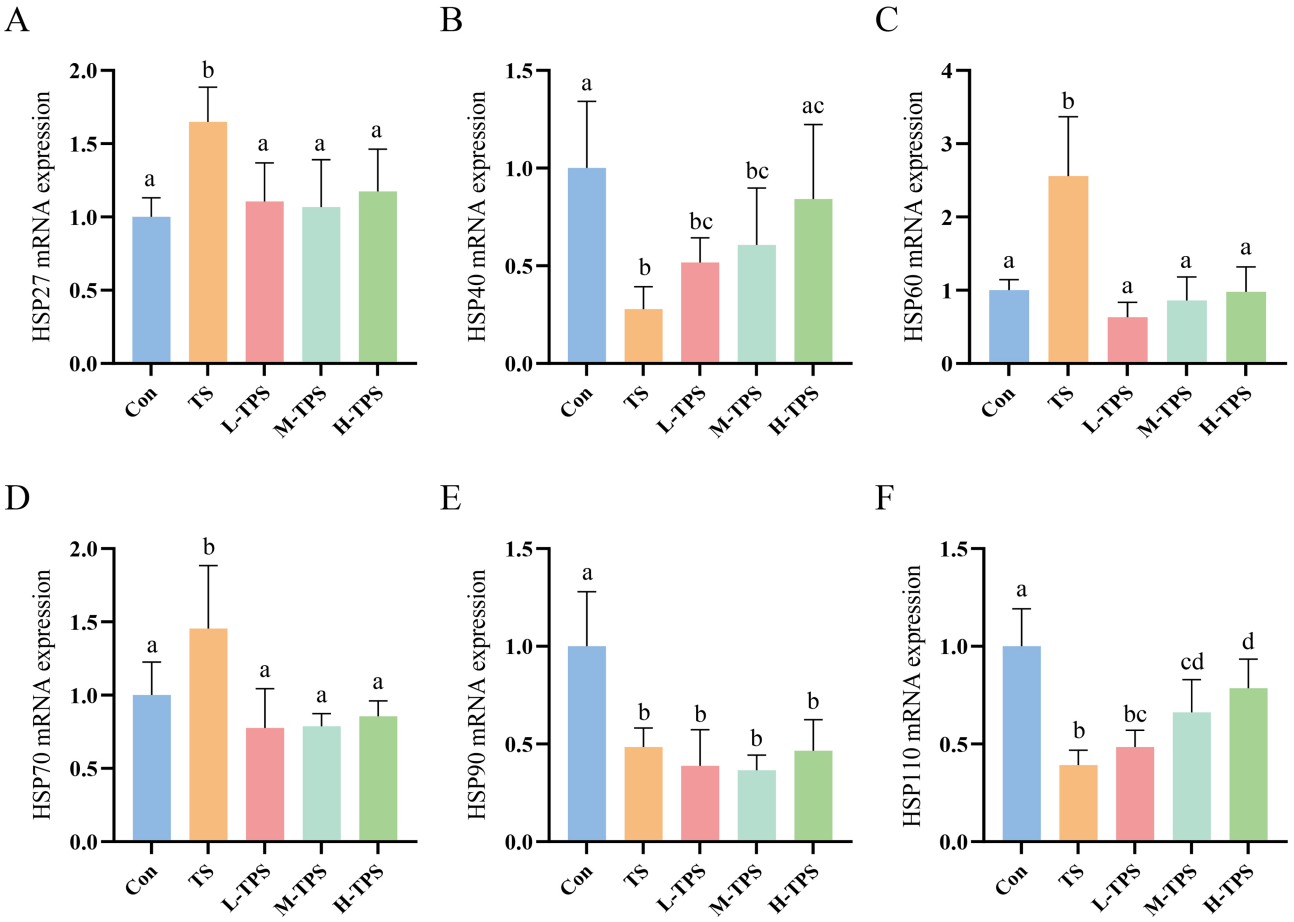
Effect of TPS on TS-induced mRNA expression levels of heat shock proteins (HSPs) in the intestine of chicks. **(A–F)** The mRNA expression levels of **(A)** *HSP27*, **(B)** *HSP40*, **(C)** *HSP60*, **(D)** *HSP70*, **(E)** *HSP90*, and **(F)** *HSP110*. Distinct letters indicate significant differences by one way analysis of variance (ANOVA) (*p* < 0.05).

As shown in Figures 8A–F, compared with the Con, the mRNA expression levels of *HSP27*, *HSP60*, and *HSP70* in the L-TPS, M-TPS, and H-TPS were significantly elevated (*p* < 0.05), whereas the mRNA expression levels of *HSP40*, *HSP90*, and *HSP110* were significantly reduced (*p* < 0.05). In comparison to the TS, the L-TPS, M-TPS, and H-TPS showed a notable reduction in the mRNA expression levels of *HSP27*, *HSP60*, and *HSP70* (*p* < 0.05). The *HSP40* mRNA expression level in the H-TPS was significantly increased (*p* < 0.05). No significant alterations were observed in the mRNA expression levels of *HSP90* in the L-TPS, M-TPS and H-TPS (*p* > 0.05). Furthermore, the mRNA expression levels of *HSP110* in the M-TPS and H-TPS were notably increased compared with the TS (*p* < 0.05).

#### 3.3.4 Effect of TPS on aquaporin in TS-induced chick intestines

**Figure 9:**
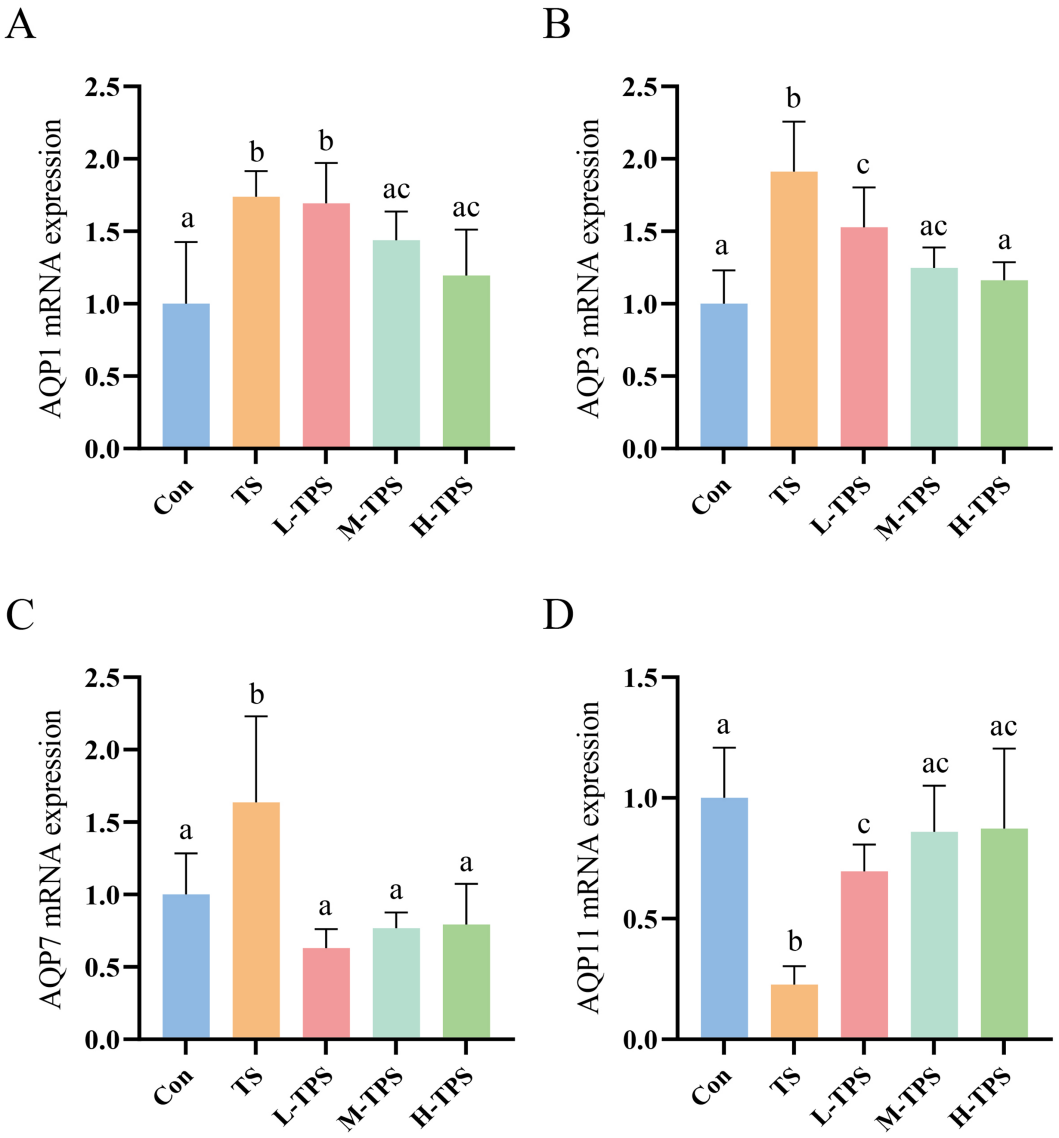
Effect of TPS on TS-induced mRNA expression levels of aquaporins (AQPs) in the intestine of chicks. The mRNA expression levels of **(A)** *AQP1*, **(B)** *AQP3*, **(C)** *AQP7* and **(D)** *AQP11*. Distinct letters indicate significant differences by one way analysis of variance (ANOVA) (*p* < 0.05).

As shown in Figures 9A–D, compared with the Con, TS caused a significant increase in the mRNA expression levels of *AQP1*, *AQP3*, and *AQP7* (*p* < 0.05), and a significant decrease in the mRNA expression of *AQP11* (*p* < 0.05). In comparison to the TS, the *AQP1* mRNA expression levels in the M-TPS and H-TPS were notably reduced (*p* < 0.05); the *AQP3* and *AQP7* mRNA expression levels in the L-TPS, M-TPS, and H-TPS were significantly decreased (*p* < 0.05); and the *AQP11* mRNA expression levels in the L-TPS, M-TPS, and H-TPS were highly significantly increased (*p* < 0.05).

#### 3.3.5 Effect of TPS on intestinal flora in TS-induced chicks

**Figure 10:**
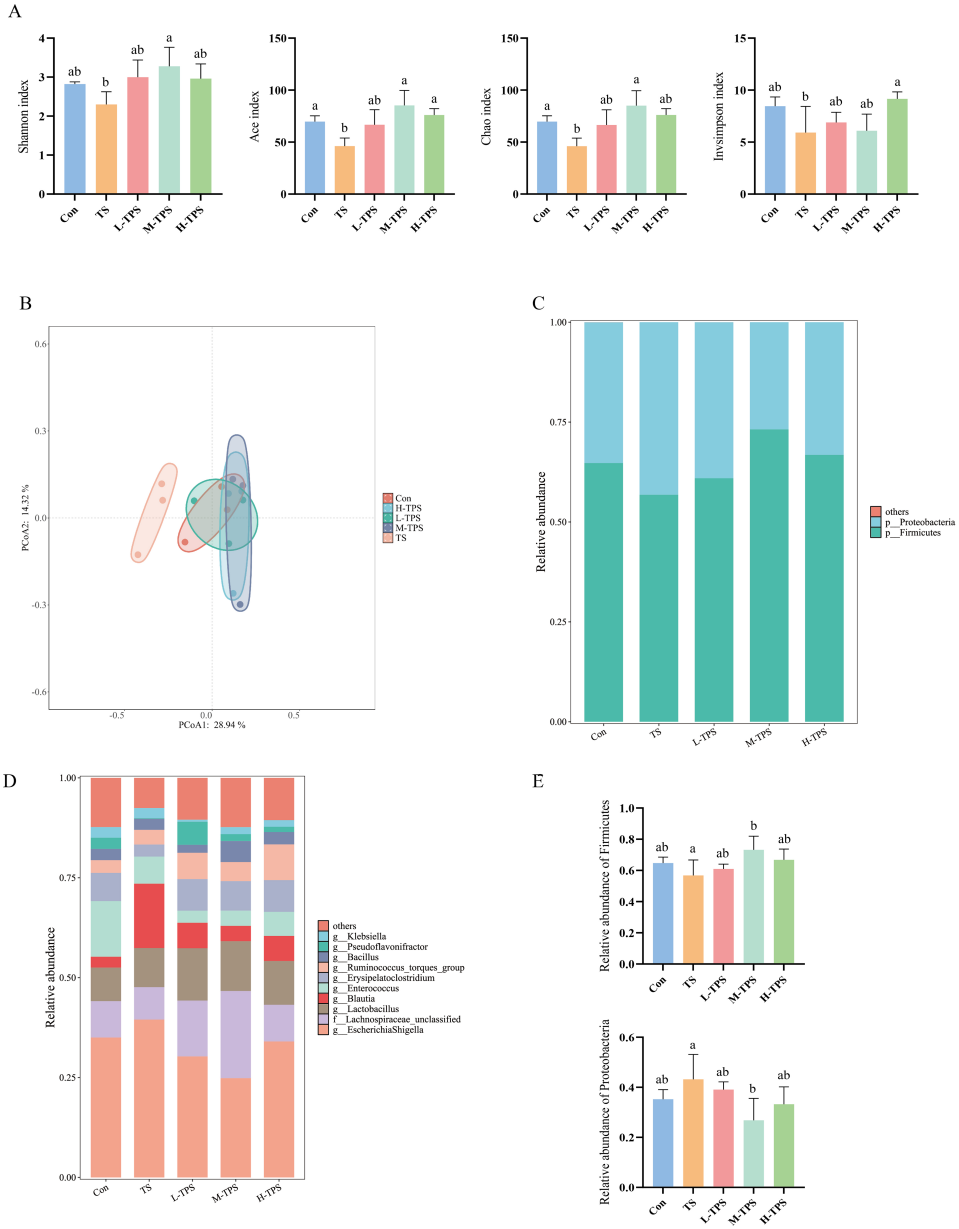
Effect of TPS on the intestinal flora in TS-induced chicks. **(A)** Alpha diversity indexes. Distinct letters indicate significant differences by one way analysis of variance (ANOVA) (*p* < 0.05). **(B)** Principal coordinate analysis (PCoA) plot. **(C)** Stacked map of species distribution at the phylum level. **(D)** Stacked map of species distribution at the genus level. **(E)** Turkey HSD test of relative abundance at the phylum level.

As illustrated in Figure 10A, in comparison to the Con, the intestinal flora in the TS showed in a notable reduction in the Ace and Chao indexes (*p* < 0.05); and a declining trend was observed in the Shannon and Invsimpson indexes (*p* > 0.05). In comparison to the TS, the Shannon, Ace, and Chao indexes were significantly elevated in the M-TPS (*p* < 0.05). Additionally, the Invsimpson and Ace indexes were significantly elevated in the H-TPS (*p* < 0.05).

In the principal coordinate analysis (PCoA, Figure 10B), PCoA1 and PCoA2 contributed 28.94 and 14.32% to the variation in the samples, respectively. The PCoA plot revealed notable discrepancies in the distances between the TS and the other groups (Con, L-TPS, M-TPS, and H-TPS). The intestinal flora in the TPS treated groups (L-TPS, M-TPS, and H-TPS) exhibited a closer proximity to the Con.

As illustrated in Figure 10C, at the phylum level, the chick cecum flora was predominantly composed of Firmicutes and Proteobacteria. In comparison to the Con, the TS exhibited a tendency to increase in the relative abundance of Proteobacteria and a tendency to decrease in the relative abundance of Firmicutes(*p* > 0.05). Compared with the TS, the M-TPS had a significantly higher relative abundance of Firmicutes and a significantly lower relative abundance of the Proteobacteria (*p* < 0.05). As illustrated in Figure 10D, at the genus level, the predominant intestinal flora observed in each group were *Escherichia Shigella*, *Lachnospiraceae_unclassified*, *Lactobacillus*, *Blautia*, *Enterococcus* and *Erysipelatoclostridium*. The relative abundance of *Escherichia Shigella* and *Blautia* exhibited a tendency to increase in the TS compared with the Con (*p* < 0.05). Compared with the TS, the relative abundances of *Escherichia Shigella* and *Blautia* a tendency to decrease in the TPS treated groups (*p* < 0.05). Notably, the relative abundance of *Lactobacillus* exhibited a tendency to increase in the TPS treated groups compared with the Con (*p* < 0.05).

## 4 Discussion

DPPH, ABTS and hydroxyl radicals are frequently utilized to evaluate antioxidant activity *in vitro*. DPPH radicals, a highly stable radical, have been extensively employed to assess the free radical scavenging capacity of compounds or extracts (23). ABTS, a water-soluble radical initiator, can be oxidized to green ABTS radicals, and this method has been widely adopted to assess the total antioxidant capacity of compounds (24). Hydroxyl radicals, the most reactive oxygen radicals, are known to be highly invasive and toxic to cells (25). In this study, the DPPH radical and hydroxyl radical scavenging capacity of TPS increased with increasing concentration, demonstrating a positive correlation. The DPPH radical scavenging capacity of TPS reached a maximum of 91.42% at 3 mg/mL, which was comparable to that of VC at the same concentration. The scavenging capacity of TPS for the ABTS radicals reached a maximum at 4 mg/mL, which was 1.87 mmol/ L. The reducing capacity of polysaccharides is also an important indicator for assessing their antioxidant capacity. In this study, the ferric ion reducing ability of TPS always showed a positive correlation with the concentration in the range of 0.5–5 mg/mL. The results indicated that TPS has good antioxidant activity.

RAW264.7 macrophages are frequently chosen as model cells to evaluate the antioxidant activity of polysaccharides (26). It has been reported that H_2_O_2_ stimulation can cause oxidative stress, resulting in damage of intracellular biomolecules, such as nucleic acids, membrane lipids, and proteins (27). Therefore, we chose H_2_O_2_ to construct the cell oxidative damage model. In the present study, TPS significantly alleviated H_2_O_2_-induced apoptosis in RAW264.7 macrophages. Further studies revealed that TPS significantly increased the activities of T-SOD, T-AOC, and GPx in the cells (*p* < 0.05). Liu et al. investigated the protective effect of Tien Shan green tea polysaccharide (TSPS) against oxidative damage in cells, finding that TSPS protected HepG2 and LO2 cells from H_2_O_2_-induced apoptosis. They also found that TSPS regulated the level of ROS and increased the activities of SOD and CAT in cells (28), which was similar to the results of the present study. A multitude of studies have demonstrated that plant polysaccharides can mitigate the damage caused by stress by modulating the antioxidant capacity of the body (29–31). Consequently, it is hypothesized that TPS with potent antioxidant activity may exert an anti-stress effect.

The transportation of animals is not only a matter of animal welfare, but also has implications for economic losses. However, because of the necessity for production and biosecurity, chicks are inevitably subjected to road transportation, which results in their exposure to TS. Various plant polysaccharides have been shown to maintain intestinal health by improving intestinal function (32, 33). In this study, shedding of intestinal villi epithelial cells occurred after 5 h of transport, while the integrity of intestinal villi was improved under TPS intervention. The villus height/crypt depth ratio is an important indicator of the digestive and absorptive function of an organism. In this study, compared with the Con, TS significantly reduced the villus height and the villus height/crypt depth ratio. However, compared with the TS, the M-TPS and H-TPS had significantly higher villus height and a larger villus height/crypt depth ratio, similar to the findings of Zou (34). The results suggested that TPS can ameliorate TS-induced intestinal damage in chicks by improving intestinal villus height and the villus height/crypt depth ratio.

The process of oxidative damage is closely related to stress. Studies have demonstrated that a variety of antioxidant-related signaling pathways, including the Nrf2 (35), PI3K/AKT (36) and NF-kB pathways (21), are activated during periods of stress. The antioxidant system of an organism consists of enzymatic and non-enzymatic systems: T-AOC reflects the cumulative effect of all antioxidants in the non-enzymatic system; the activities of GPx and T-SOD reflect the status of the enzymatic system; and the content of MDA reflects the degree of lipid oxidation of the cells. In this study, the T-AOC, T-SOD and GPx levels were significantly decreased, and the content of MDA was significantly increased in the TS, suggesting that oxidative damage occurred in the jejunum. Surprisingly, T-SOD, T-AOC, GPx, and MDA levels were restored to normal levels after TPS intervention. Liu found that Tien Shan green TPS could protect cells from H_2_O_2_ damage by activating the Nrf2 signaling pathway (28). Therefore, we speculated that TPS might alleviate TS-induced oxidative damage by activating the Nrf2 pathway in intestinal tissues, which requires further investigation.

HSR is one of the most conserved pathways in organisms when confronted with stress and unfavorable conditions. It is a classical cellular response to maintain the accurate folding of proteins and to prevent the aggregation of erroneous proteins from interfering with the cell’s normal physiological functions. In eukaryotes, HSR is regulated by heat shock factors (including HSF1-4) (37). *HSF1* plays a crucial role in antagonizing environmental perturbations and proteotoxic stresses (38). In this study, TS induced a significant increase in *HSF1* mRNA expression, which regulated the expression of heat shock proteins (HSPs). In recent years, *HSF2* has been shown to co-regulate the expression of HSPs with *HSF1* (39), and thus the expression of *HSF1* and *HSF2* tended to occur the same direction after TS in the chick jejunum. *HSF3* is essential for the HSR in avian. It has been demonstrated that the *HSF3* has a higher threshold and increased expression during severe heat shock (40). In this study, the expression of *HSF3* was significantly elevated in the TS, indicating that the TS applied to the chicks in this experiment reached the threshold for *HSF3* activation. The notable decline in *HSF3* expression following TPS intervention indicated that TPS significantly mitigated the adverse effects of TS on the chicks.

HSPs are the main executors of the HSR. However, excessive stress can cause HSPs to lose their protective effects (41). Sun found that the expression of HSPs in chick hearts was elevated in the early stage of TS. However, most of the HSPs were reduced as TS was intensified (4). Therefore, the expression levels of HSPs might be related to the degree of stress. In this study, the mRNA expression levels of *HSP27*, *HSP60*, and *HSP70* increased, and those of *HSP40*, *HSP90*, and *HSP110* decreased in response to TS. The results indicated that transportation for 5 h induced a severe stress response in the chick intestines, which was also evidenced by the expression of *HSF3*. Compared with the TS, the mRNA expression levels of *HSP27*, *HSP60*, *HSP70* were significantly decreased in TPS treated groups, and the mRNA expression levels of *HSP40* and *HSP110* were significantly increased. The results indicated that TPS could restore the HSR in the jejunum of chicks that was impaired by TS.

AQPs are widely distributed in a wide range of tissues. They maintain organismal homeostasis mainly by regulating the intra- and extracellular transport of water molecules and small solutes (42). AQPs are functionally classified into three distinct groups: Firstly, AQP1, 2, 4, and 5, which primarily transport water molecules; secondly, a group of aquaglyceroporins, including AQP3, 7, 9, and 10, which transport water, glycerol, and other small solutes; and thirdly, a group including AQP6, 8, 11, and 12, which have a variety of specific cellular localizations and functions (43, 44). In this study, the mRNA expression levels of *AQP1*, *AQP3*, and *AQP7* were significantly increased, while the expression of *AQP11* mRNA was significantly decreased in the intestines of chicks exposed to TS. Wang found that AQPs mRNA levels were significantly increased in the intestines of pyrexic rats, which is consistent with our findings (45). In comparison to the TS, the mRNA expression levels of *AQP1*, *AQP3*, and *AQP7* were significantly decreased, while the expression level of *AQP11* mRNA was significantly increased in the TPS treated groups. These findings suggested that TPS can antagonize the TS-induced intestinal homeostatic imbalance and might serve as a potential therapeutic agent to treat TS-induced intestinal homeostatic imbalance.

The intestinal flora plays a pivotal role in the growth, development, immune function, and metabolism of animals (46). Consequently, an increasing number of studies investigating the function of plant polysaccharides are considering the intestinal flora as a crucial target. Polysaccharides have been shown to enhance intestinal health by inhibiting the proliferation of detrimental bacteria, promoting the growth of beneficial bacteria, and strengthening the intestinal mucosal barrier (47). In this study, 16S rDNA high-throughput sequencing was employed to investigate the impact of TPS on the intestinal flora of TS-treated chicks. The findings indicated a decline in the alpha diversity of the intestinal flora of chicks subjected to TS, accompanied by alterations in beta diversity. However, after TPS intervention, alpha diversity was significantly higher and beta diversity was normalized. At the phylum level, the dominant intestinal flora of chicks was identified as Firmicutes and Proteobacteria. A reduction in the abundance of Firmicutes and an increase in Proteobacteria were observed in the TS, indicating that TS disrupted the intestinal flora structure of the chicks, which is in line with the findings of Zhao (48). However, the abundance of Firmicutes increased and the abundance of Proteobacteria decreased following TPS intervention. At the genus level, the administration of TS resulted in an increase in the relative abundance of *Escherichia Shigella* and *Blautia* in the chick. It has been found that stress can increase the level of inflammation, which in turn disrupts the structure of the intestinal flora, resulting in a decrease in the relative abundance of Firmicutes and an increase in the relative abundance of *Blautia* and *Ruminococcaceae* in the intestines of mice (49), which is similar to our findings. Therefore, we hypothesized that TS might cause an inflammatory response in the intestine of chicks, which in turn leads to disruption of the intestinal flora. The relative abundance of *Escherichia Shigella* and *Blautia* was reduced after TPS intervention. In addition, the relative abundance of *Lactobacillus*, a beneficial bacterium, increased in the TPS treated groups. The experimental results showed that TS could disrupt the intestinal flora of chicks, whereas TPS could maintain intestinal health by regulating the structure and composition of the intestinal flora.

## 5 Conclusion

In conclusion, the present study demonstrated that TPS extracted from Jinxuan tea has good antioxidant activity and can effectively alleviate H_2_O_2_-induced cellular oxidative damage. A transport stress model was established, and it was found that TS induces oxidative damage, disrupts the HSR and the expression of AQPs in the chick intestine, while also influencing the structure and composition of the intestinal flora. However, TPS was demonstrated to mitigate the oxidative damage, increase the intestinal flora abundance, and modulate the HSR and AQPs expression, thereby mitigating intestinal damage. The present study increases our understanding of intestinal damage in chicks caused by TS and provides a basis for the development and utilization of TPS as an anti-stress feed additive.

## Data Availability

The raw data supporting the conclusions of this article will be made available by the authors, without undue reservation.
